# A decade of arteriovenous fistula creations in the ⩾75 years population: Equal opportunity or sub-optimal use of resources

**DOI:** 10.1177/11297298221147571

**Published:** 2023-01-06

**Authors:** Michael Corr, Astha Pachchigar, Michael O’Neill, Rebecca Higgins, Stephen O’Neill, Jennifer Hanko, Agnes Masengu

**Affiliations:** 1 Centre of Public Health, Queen’s University, Belfast, UK; 2 Regional Nephrology & Transplant Unit, Belfast Health and Social Care Trust, Belfast, UK; 3 School of Medicine, Queen’s University, Belfast, UK

**Keywords:** Dialysis, dialysis access, AV fistula, catheters, ultrasonography—Doppler evaluation, economics and health services

## Abstract

**Background:**

The optimal vascular access in the elderly remains contentious in the context of increasingly limited resources and anticipated survival on hemodialysis. Research focus has shifted to include the impact of vascular access on quality of life. This study explored clinical outcomes in individuals aged ⩾75 years who had an arteriovenous fistula (AVF) created in a single center over a 10-year period.

**Materials and methods:**

Demographic and clinical data concerning AVFs created January 2009–December 2019 were identified from a prospective database for retrospective analysis. Outcome measures were AVF patency and failure to mature rates plus overall patient and vascular access survival. The Vascular Access Specific Quality of life measure (VASQoL) was completed in a contemporary cohort aged ⩾75 years established on HD in October 2021.

**Results:**

AVF outcomes were available for 272 patients (93%). The failure to mature (FTM) rate was 36% with the significant predictors of AVF FTM being the creation of a radiocephalic AVF (OR 8.13, 95% CI 8.02–8.52, *p* < 0.01), female gender (OR 4.84, 95% CI 4.70–5.41, *p* < 0.01), and a history of peripheral vascular disease (OR 5.25, 95% CI 5.22–6.00, *p* value = 0.02). Functional patency was associated with a median 12-month survival benefit compared to those whose fistula FTM (*p* < 0.01). The median patency duration for a functionally patent AVF was 3 years. Elderly patients with a fistula reported a lower quality of life in VASQoL scoring than those with central venous catheters.

**Conclusions:**

In this cohort, AVF creation in individuals aged ⩾75 years AVFs was associated with comparable AVF patency rates to younger patients. AVF functional patency was associated with superior patient survival compared to those with AVF FTM. A multi-disciplinary surveillance program may help reduce AVF loss. Further work on how vascular access choice impacts quality of life in elderly patients is required.

## Background

End-stage renal disease (ESRD) is increasing with chronic kidney disease (CKD) set to be the fifth leading cause of death worldwide by 2040.^1^ In the United States and the United Kingdom, the highest increase in ESRD incidence is in the ⩾75-year-old population.^2,3^ The majority of these individuals (>90%) will commence hemodialysis (HD) as their incident renal replacement therapy (RRT) modality and very few will subsequently switch to peritoneal dialysis or receive a kidney transplant.^2^ The rise in incident HD in the ⩾75-years population has resulted in significant increases in the prevalent HD populations in most healthcare settings.^4^

For the general HD population, the associated survival benefits of arteriovenous fistula (AVF) use for vascular access are well established.^5^ However, in the ⩾75 years age category, the benefits are less clear. This cohort have increased morbidities such as significant vascular disease which may predispose to higher AVF failure to mature (FTM) rates and, it has been suggested, require a longer maturation time before successful needling of the AVF.^6,7^ Surgical complications including wound infection and AVF thrombosis are also reported to be more common in those ⩾75 years.^8^ In the setting of cognitive impairment, patients may not be able to accurately follow post-operative AVF care instructions and exercises that may enhance successful maturation^9^; potentially making the time between formation of a new AVF and initiation of HD even more complicated with increased risk of bridging access with a HD central venous catheter (CVC) (and its associated morbidity and mortality).^10^ Added to this is the limited life-expectancy of HD individuals aged ⩾75 years old.^11^

Waiting lists for vascular access related elective surgery and interventional radiology commonly outstrip demand. Challenges regarding access to elective theater lists have been amplified by the COVID-19 pandemic^12^ with HD related vascular access surgical services relegated much further down than ladder making it an even more precious resource. There is conflicting evidence of how AVF impacts the quality of life of patients, particularly in older cohorts, requiring introspection of whether the service is appropriate for elderly patients with ESRD.^13^

The primary aim of this study was to retrospectively analyze the clinical outcomes in persons aged ⩾75 years who had an AVF created in a single European center from January 2009 to December 2019. The secondary aim was to assess the differences in quality of life between contemporaneous ⩾75 patients with CVC or AVF.

## Methods

### Study design

This was a single-center retrospective cohort study using a prospectively designed clinical database—The Northern Ireland Vascular Access Database for Chronic Kidney Disease (ethics approval reference number 20/NI/0069). The database prospectively collects clinical and vascular access data of patients with advanced chronic kidney disease. Recorded patient characteristics include gender, age, race, ethnicity, primary renal disease, type of vascular access, pre-operative ultrasound measurements, co-morbidities, RRT status, patient survival, and access survival data.

### Study setting and population

The study was set in the Regional Nephrology and Transplant Unit—Belfast Health and Social Care Trust, Northern Ireland. The center provides dialysis access procedures for the whole of Northern Irish population. The pre-operative duplex ultrasound examination for vascular mapping was performed by a single practitioner (J.H.) using a Sonosite M-Turbo ultrasound machine (Sonosite, Bothell, Wash) with a high frequency (13–6 MHz) linear probe. All patients for consideration of HD receive vascular mapping with the only absolute contraindication to referral being patient choice. Following mapping, clinical assessment and discussion with patient, if suitable, a tailored recommendation of AVF site is made. The AVF surgery was undertaken by six senior surgeons who specialize in renal access and transplant surgery. The service is supported by a vascular access specialist nursing team and an interventional radiology team with senior radiologists experienced in vascular access.

The study population included all patients aged ⩾75 years who had an AVF created between January 2009 and December 2019 and in whom the AVF had a functional outcome recorded by September 2021.

### Data collection

Key demographic data collected from the clinical database included age, gender, race, primary renal disease, comorbidities. Renal specific data was also collected including renal replacement therapy (RRT) status at the time of AVF creation, HD start date, previous access attempts, and AVF outcomes.

Fistula characteristics were also scrutinized from the database including site, laterality and whether ultrasonographic mapping had occurred.

AVF outcome measures were defined as;

- Functional patency: Two-needle use on HD for at least six consecutive hemodialysis sessions (including AVFs that achieved assisted patency).
- Primary patency: Two-needle use on HD for at least six consecutive sessions without intervention after AVF creation.
- Primary assisted patency: An AVF that required surgical or radiologic intervention after initial creation before functional patency.
- FTM was defined by clinical assessment, ultrasound assessment or abandonment of the AVF following failure to sustain functional patency.

Vascular access specific Patient Reported Outcome Measures (PROMS) have been recommended by Kidney Health Initiative to enable the patient voice to be heard.^14^ The Vascular Access Specific Quality of life measure (VASQoL)^15^ is a self-administered 11 item questionnaire that was completed by a cohort of individuals aged ⩾75 years established on HD in October 2021 to provide additional information on patient experience and satisfaction with an AVF compared to a CVC.

### Statistical analysis

Statistical analysis was performed using SPSS Statistics version 24 (IBM Corp, Armonk,NY). Logistic regression was used to identify variables of statistical significance that predicted AVF FTM. The Independent t-test was used to compare baseline clinical outcomes between the primary (and assisted primary) patent and FTM groups.

## Results

A total of 1032 individuals had an AVF created between January 2009 and December 2019, 297 individuals were aged ⩾75 years created representing 27% of total AVF creations. A functional outcome was available for 272 patients (93%) with the remaining being lost to follow-up. The clinical details are summarized in Table 1. The median age at fistula creation was 80 years old. There was a predominance for males (67%) and the population was almost exclusively Caucasian in-keeping with the locality of the study.

**Table 1.** Clinical characteristics of 297 individuals aged ⩾75 years who had 319 AVFs created between January 2009 and December 2019.

Clinical characteristics	Number (%)
Age (years); median; range	80 (75–93)
Gender	
Male	200 (67)
Female	97 (33)
Ethnicity	
White	296 (99.7)
Non-White	1 (0.3)
Co-morbidities	
Diabetes	92 (31)
Peripheral vascular disease	19 (6)
Coronary artery disease	124 (42)
CKD status as AVF creation	
Pre-dialysis	211 (66)
Hemodialysis	104 (33)
Peritoneal dialysis	4 (0.1)
Formal peri-operative ultrasound assessment^a^	
No	175 (55)
Yes	144 (45)
Location of AVF	
Forearm	125 (39)
Upper arm	194 (61)

a Formal peri-operative mapping was established in 2012.

Of the 272 patients with functional outcomes, three had early technical failure and two were ligated due to development of steal syndrome. For the remaining 267 patients, 171/267 (64%) achieved functional AVF patency (primary patency = 145, primary assisted patency = 26) whilst 96/267 (36%) had AVF FTM. About 62% of the patients with primary assisted patency had forearm AVFs.

The results of the logistic regression analysis of multiple clinical variables exploring predictive variables for AVF FTM is shown in Table 2. Of the variables measured, forearm in comparison to upper arm AVF (OR 8.13, 95% CI 8.02–8.52, *p* < 0.01) and female gender (OR 4.84, 95% CI 4.70–5.41, *p* < 0.01) were strongly associated with FTM. A past medical history of peripheral vascular disease was also associated with increased risk of FTM (OR 5.25, 95% CI 5.22–6.00, *p* < 0.05).

**Table 2.** Logistic regression analysis of predictors of AVF FTM.

Clinical variable	Odds ratio	95% Cl	*p* Value
Female gender	4.84	4.70–5.41	<0.01
On HD prior to creation	2.44	2.10–3.57	0.11
Not US mapped	2.12	1.65–3.81	0.73
Lower forearm	8.13	8.02–8.52	<0.01
Diabetes mellitus	2.01	1.71–3.22	0.16
Peripheral vascular disease	5.25	5.22–6.00	0.02
Ischemic heart disease	1.34	0.57–4.17	0.25

US: ultrasound.

Pre-operative ultrasound vessel assessment was performed for 144 (49%) of individuals. The use of perioperative ultrasound assessment was not found to be predictive of FTM in this cohort. Interestingly, if the optimal AVF creation site recommended on ultrasound mapping was created by the surgeon this increased the odds of primary patency by 75.8% (*p*-value <0.01). In the 26/144 cases where a different AVF was created than recommended by ultrasound assessment, the functional patency rate was 46% compared to 82% (different AVF creation site versus site recommended on mapping).

About 15.2% of patients with a functional AVF in our cohort required intervention prior to first use (primary assisted patency). Radiological intervention was required for 26% of patients to maintain the functioning of their AVF from creation until conclusion of the study in September 2021. The mean number of interventions for this group was 1.8 with a range of 1–5.

The clinical outcomes of all 293 patients by September 2021 were obtained and can be viewed in Table 3.

**Table 3.** Patient outcomes at the end of the observation period (*n* = 297).

Patient outcomes at the end of the observation period	Number (%)
Deceased	220 (74)
Transplanted	4 (1)
Hemodialysis	67 (23)
CKD	6 (2)

Three quarters of patients, 220/293 (74%), were deceased at the end of the follow up period reflecting the age, duration of study and prognosis on HD. The median survival for individuals who survived the first year of dialysis (78%) was 5 years (entire cohort 4.8 years). The median survival of a functional AVF was 3 years. Of the 171 AVFs that achieved functional patency 18 thrombosed, 10 were ligated (six steal, three bleeding, one lower arm fracture fixation), eight were subsequently abandoned (steal, prolonged bleeding), and two patients declined use due to pain when needling.

In this cohort, AVF patency was associated with increased survival. The mean time from AVF creation to death in the functionally patent AVF group was 3.4 years compared to 2.6 years in the AVF FTM group (*p*-value 0.002). Twenty-seven patients (9%) had an AVF created that was subsequently never used as they did not require HD before their death.

Of the 269 individuals established on dialysis in this cohort, 122 (45%) started dialysis with a CVC. Patients who commenced HD with a functioning AVF had a mean survival of 3.8 years compared to those who commenced dialysis with a CVC then converted to AVF who had a mean survival of 2.8 years. However, this was not statistically significant (*p* value = 0.15).

Individuals aged 75 or older dialyzing in the Belfast Trust Renal Unit in October 2021 were invited to complete the VASQoL questionnaire. In the renal unit a total of 132 patients attend outpatient HD, 42 patients were aged 75 or over. Partaking in the questionnaire was optional and reason for patient refusal was not documented. Any patients who wished to take part but were limited by a disability, such as visual impairment, were assisted by a member of staff. Eighteen patients with AVF and 16 patients with a CVC completed the questionnaire; their demographic details can be viewed in Table 4. Of the 18 patients surveyed with AVF; 16 patients had primary patency and two patients had primary assisted patency. Five of the patients with primary patency had received interventions following maturation to maintain AVF adequacy. In the CVC cohort, six patients had >2 tunneled line insertions, a single patient had line related sepsis.

**Table 4.** Characteristics of individuals aged ⩾75 years (*n* = 34) who completed the VASQoL questionnaire.

Characteristic	AVF cohort (*n* = 18)	CVC cohort (*n* = 16)
Age; mean (range)	80 years (75–90)	80 years (75–86)
Gender (*n*)		
Male	16	12
Female	2	4
Mean duration of HD (years)	4.5	2.5
Assistance with PADL^a^ (%)	17	31
Use of walking aid (%)	67	63
Able to climb stairs (%)	67	44

a Personal activities of daily living-bathing/showering, dressing, personal hygiene and grooming, bladder and bowel management, feeding.

The AVF and CVC groups reported similar amounts of satisfaction with the appearance and feel of their vascular access. About 17% of individuals with an AVF reported moderate concern about complications such as thrombosis, failure or infection compared to 13% in the CVC group.

About 33% of individuals with an AVF reported moderate to high interference of their AVF in relation to activities of enjoyment or interference with relationships. About 94% of individuals with a CVC felt included in decisions regarding their access compared to 72% with an AVF. When asked about levels of satisfaction with life in general 55.6% in the AVF group and 62.5% in the CVC group reported that their satisfaction levels were high.

## Discussion

Optimal management of vascular access in those aged ⩾75 years remains controversial. Our data relates to a real world European cohort who had all been selected as potential candidates for AVF creation (reducing selection bias). This study suggests a survival benefit in those patients with successful AVF creation over those with FTM and subsequent CVC use. These findings would be in keeping with other observational studies of the general HD population^10,16–18^ and in those ⩾75 years.^19^ However, observational data comparing mortality in patients with CVC versus AVF may be confounded by selection bias as those dialyzing with a CVC are likely to sicker and frailer. Recent studies have suggested that CVC associated mortality has been historically overestimated.^20^ One study demonstrated no difference in mortality between those with successful and unsuccessful AVF creation, suggesting selection for AVF rather than AVF outcome was a predictor for mortality.^21^ If there is indeed any survival benefit in those ⩾75s years it is likely to be even more marginal than in the general HD population hence why updated international guidelines on vascular access have suggested a more nuanced approach to AVF creation.^22^

Reported FTM rates in different centers range significantly in the ⩾75s which could be attributable to population characteristics, patient selection, and surgical approaches used.^22–24^ There are high rates of renal transplantation in Northern Ireland (80 per million of population) with 11% of patients transplanted annually being ⩾70 years.^25^ PD use has also traditionally been lower than the rest of the UK (e.g. 49 per million population vs 72 per million population in England).^25^ This means that the HD population at our center tends to be particularly frail. Despite this the FTM rate in this cohort was lower than in all age groups reported from our center in 2009 to 2015 (mean age 65 years) (FTM rates 36% vs 41%).^26^ There are likely many attributable reasons for this improvement in outcomes but we feel careful patient selection and AVF planning by a dedicated vascular access team are the major contributory factors.

Whilst usability for dialysis is an important outcome in measuring success of maturation, how the fistula became usable is an important factor to consider. About 15.2% of patients with a functional AVF in our cohort required intervention prior to first use and 26% of functional AVFs required further intervention to maintain function. These rates are low compared to other observational studies^27–29^ but the effect on the patient experience and the economic burden should not be underestimated.

The site of AVF creation is an important predictive variable for rates of AVF FTM. In our unit, in the older individual with a more limited life expectancy, we adopt a nuanced approach, “right patient, right access creation for success at first attempt.” Reflecting this, 61% of patients in our cohort had an upper limb AVF creation. In this study, a forearm AVF creation was strongly associated with increased FTM risk (OR 9.13). Upper limb AVF creation in the elderly and the reduced emphasis on “prioritize distal” in access creation guidelines is an increasingly common and successful access strategy.^30,31^

Arhuidese et al.^19^ published a large retrospective analysis of vascular access for elderly patients requiring HD in the United States. They recommended that AVF should be considered in elderly dialysis patients with a life expectancy over 4 months and who can tolerate surgery. However, tolerability of surgery might soon become a moot consideration with the advent of increasingly sophisticated surgical and interventional approaches such as percutaneous endovascular AVF formation.^32,33^ All procedures carried out in our study were completed under local anesthetic reducing risk from general anesthesia whilst only experiencing three early technical failures. Furthermore, assessment of frailty in potential vascular access and HD patients is limited by the lack of validated frailty assessment scores for this population.^34^ Studies have found that nephrologists’ perceived frailty as a surrogate for formal assessment of HD patients is poor.^35^ Until further work is done to validate clinical frailty scores in the HD population and how it affects vascular access planning,^36^ patient-centered outcomes such as quality of life could perhaps offer a better alternative.

Our data from the VASQoL assessment tool raises important questions of how elderly patients view their AVFs and how it affects their quality of life. It is concerning that AVF patients reported lower quality of life than those with CVC and felt less involved in their vascular access care. Our results however have to be taken in the context of patients with AVF being on HD for a longer period of time than those with CVC and the lack of assessment for confounders such as depression in these groups. Assessment of how AVF creation, maintaining function and repeated use for HD, impacts elderly patients’ quality of life in an underexplored area in the literature. Further work in this field may help inform clinical practice and patient selection for AVF creation. Understanding better the subjective patient reality of different vascular access options will also assist guide clinicians and patients in shared decision making.

### Limitations

This is a single-center retrospective observational study which limits the interpretation and generalizability of our findings. However, it presents 10 years data from an established European AVF cohort in a center with substantial vascular access expertise which adds further information to this understudied area.

## Conclusions

In our cohort those patients ⩾75 years who had an AVF that achieved functional patency had a survival benefit over those with AVF FTM. As within the wider literature; it remains unclear whether this survival benefit is due to patients having a functional AVF or due to other health parameters as no causal relationship has been demonstrated. Female gender, forearm creation site and history of peripheral vascular disease were associated with FTM. 15% of functional AVFs required intervention prior to use and 26% required intervention to maintain functionality. Careful patient selection and choosing the most likely site for successful AVF creation (upper arm) for AVF creation may reduce FTM and intervention rates in the elderly population. Further work on how AVF affects quality of life in elderly patients is required.

## References

[1] Foreman KJ Marquez N Dolgert A , et al. Forecasting life expectancy, years of life lost, and all-cause and cause-specific mortality for 250 causes of death: reference and alternative scenarios for 2016–40 for 195 countries and territories. Lancet 2018; 392(10159): 2052–2090. 30340847 10.1016/S0140-6736(18)31694-5 PMC6227505

[2] United States Renal Data System. USRDS annual data report: epidemiology of kidney disease in the United States. Bethesda, MD: National Institutes of Health, National Institute of Diabetes and Digestive and Kidney Diseases, 2021.

[3] UK Renal Registry. UK renal registry 23rd annual report – data to 31/12/2019, Bristol, UK, renal.org/audit-research/annual-report (2021). (accessed May 2022).

[4] Liyanage T Ninomiya T Jha V , et al. Worldwide access to treatment for end-stage kidney disease: a systematic review. Lancet 2015; 385(9981): 1975–1982. 25777665 10.1016/S0140-6736(14)61601-9

[5] Gallieni M Hollenbeck M Inston N , et al. Clinical practice guideline on peri- and postoperative care of arteriovenous fistulas and grafts for haemodialysis in adults. Nephrol Dial Transplant 2020; 35(12): 2203. 10.1093/ndt/gfaa106 32365363

[6] Lee T Thamer M Zhang Y , et al. Outcomes of elderly patients after predialysis vascular access creation. J Am Soc Nephrol 2015; 26(12): 3133–3140. 25855782 10.1681/ASN.2014090938 PMC4657836

[7] Woo K Goldman DP Romley JA. Early failure of dialysis access among the elderly in the era of fistula first. Clin J Am Soc Nephrol 2015; 10(10): 1791–1798. 26254301 10.2215/CJN.09040914 PMC4594075

[8] Borzumati M Funaro L Mancini E , et al. Survival and complications of arteriovenous fistula dialysis access in an elderly population. J Vasc Access 2013; 14(4): 330–334. 23599142 10.5301/jva.5000143

[9] Kurella Tamura M Yaffe K . Dementia and cognitive impairment in ESRD: diagnostic and therapeutic strategies. Kidney Int 2011; 79(1): 14–22. 20861818 10.1038/ki.2010.336 PMC3107192

[10] Xue JL Dahl D Ebben JP , et al. The association of initial hemodialysis access type with mortality outcomes in elderly medicare ESRD patients. Am J Kidney Dis 2003; 42(5): 1013–1019. 14582045 10.1016/j.ajkd.2003.07.004

[11] Chan KE Maddux FW Tolkoff-Rubin N , et al. Early outcomes among those initiating chronic dialysis in the United States. Clin J Am Soc Nephrol 2011; 6(11): 2642–2649. 21959599 10.2215/CJN.03680411 PMC3359565

[12] COVIDSurg Collaborative. Projecting COVID-19 disruption to elective surgery. Lancet 2022; 399(10321): 233–234. 34922640 10.1016/S0140-6736(21)02836-1 PMC8676422

[13] Auguste BL Chan CT. Home dialysis among elderly patients: outcomes and future directions. Can J Kidney Health Dis 2019; 6: 2054358119871031. 31523436 10.1177/2054358119871031 PMC6732853

[14] Field M Tullett K Khawaja A , et al. Quality improvement in vascular access: the role of patient-reported outcome measures. J Vasc Access 2020; 21(1): 19–25. 31081441 10.1177/1129729819845624

[15] Richarz S Greenwood S Kingsmore DB , et al. Validation of a vascular access specific quality of life measure (VASQoL). J Vasc Access. Epub ahead of print 5 October 2021. DOI: 10.1177/11297298211046746. 34608832

[16] Allon M Daugirdas J Depner TA , et al. Effect of change in vascular access on patient mortality in hemodialysis patients. Am J Kidney Dis 2006; 47(3): 469–477. 16490626 10.1053/j.ajkd.2005.11.023

[17] Lacson E Jr Wang W Lazarus JM , et al. Change in vascular access and mortality in maintenance hemodialysis patients. Am J Kidney Dis 2009; 54(5): 912–921. 19748717 10.1053/j.ajkd.2009.07.008

[18] Polkinghorne KR McDonald SP Atkins RC , et al. Vascular access and all-cause mortality: a propensity score analysis. J Am Soc Nephrol 2004; 15(2): 477–486. 14747396 10.1097/01.asn.0000109668.05157.05

[19] Arhuidese IJ Cooper MA Rizwan M , et al. Vascular access for hemodialysis in the elderly. J Vasc Surg 2019; 69(2): 517–525.e1. 10.1016/j.jvs.2018.05.219 30683199

[20] Quinn RR Oliver MJ Devoe D , et al. The effect of predialysis fistula attempt on risk of all-cause and access-related death. J Am Soc Nephrol 2016; 28(2): 613–620. 28143967 10.1681/ASN.2016020151 PMC5280018

[21] Brown RS Patibandla BK Goldfarb-Rumyantzev AS. The survival benefit of “fistula first, catheter last” in hemodialysis is primarily due to patient factors. J Am Soc Nephrol 2017; 28(2): 645–652. 27605542 10.1681/ASN.2016010019 PMC5280011

[22] Lok CE Huber TS Lee T , et al.; National Kidney Foundation. KDOQI clinical practice guideline for vascular access: 2019 update. Am J Kidney Dis 2020; 75(4 Suppl 2): S1–S164. 10.1053/j.ajkd.2019.12.001 32778223

[23] Meena P Bhargava V Sehrawat S , et al. Stretching the boundaries: suitability of an arteriovenous fistula in elderly patients on hemodialysis-a northern India experience. Int Urol Nephrol 2022; 54(3): 671–678. 34244917 10.1007/s11255-021-02941-4

[24] Medricka M Janeckova J Jarosciakova J , et al. Creation of arteriovenous fistula for hemodialysis in the older population. Biomed Pap Med Fac Univ Palacky Olomouc Czech Repub 2021; 165(2): 179–183. 32285849 10.5507/bp.2020.013

[25] Annual Report on Kidney Transplantation 2020/21. NHS blood and transplant (accessed 19 February 2022).

[26] Masengu A Maxwell AP Hanko JB. Investigating clinical predictors of arteriovenous fistula functional patency in a European cohort. Clin Kidney J 2016; 9(1): 142–147. 26798475 10.1093/ckj/sfv131 PMC4720209

[27] Lee T Qian J Thamer M , et al. Tradeoffs in vascular access selection in elderly patients initiating hemodialysis with a catheter. Am J Kidney Dis 2018; 72(4): 509–518. 29784614 10.1053/j.ajkd.2018.03.023 PMC6153087

[28] Lee T Ullah A Allon M , et al. Decreased cumulative access survival in arteriovenous fistulas requiring interventions to promote maturation. Clin J Am Soc Nephrol 2011; 6(3): 575–581. 21088288 10.2215/CJN.06630810 PMC3082416

[29] Dember LM. Fistulas first – but can they last? Clin J Am Soc Nephrol 2011; 6(3): 463–464. 21393484 10.2215/CJN.00960111

[30] Pisoni RL Zepel L Fluck R , et al. International differences in the location and use of arteriovenous accesses created for hemodialysis: results from the dialysis outcomes and practice patterns study (DOPPS). Am J Kidney Dis 2018; 71(4): 469–478. 29198387 10.1053/j.ajkd.2017.09.012

[31] Peterson WJ Barker J Allon M. Disparities in fistula maturation persist despite preoperative vascular mapping. Clin J Am Soc Nephrol 2008; 3(2): 437–441. 18235150 10.2215/CJN.03480807 PMC2390953

[32] Mallios A Fonkoua H Allouache M , et al. Percutaneous arteriovenous dialysis fistula. J Vasc Surg 2020; 71(4): 1395. 32204843 10.1016/j.jvs.2020.01.034

[33] Mallios A Bourquelot P Franco G , et al. Midterm results of percutaneous arteriovenous fistula creation with the ellipsys vascular access system, technical recommendations, and an algorithm for maintenance. J Vasc Surg 2020; 72(6): 2097–2106. 32276012 10.1016/j.jvs.2020.02.048

[34] Kuningas K Inston N. Age is just a number: is frailty being ignored in vascular access planning for dialysis? J Vasc Access 2022; 23: 192–197. 33522351 10.1177/1129729821989902

[35] Salter ML Gupta N Massie AB , et al. Perceived frailty and measured frailty among adults undergoing hemodialysis: a cross-sectional analysis. BMC Geriatr 2015; 15: 52. 25903561 10.1186/s12877-015-0051-y PMC4428253

[36] Woo K Gascue L Norris K , et al. Patient frailty and functional use of hemodialysis vascular access: a retrospective study of the US renal data system. Am J Kidney Dis 2022; 80(1): 30–45. 34906627 10.1053/j.ajkd.2021.10.011 PMC9187779

